# Old and Novel Therapeutic Approaches in the Management of Hyperglycemia, an Important Risk Factor for Atherosclerosis

**DOI:** 10.3390/ijms23042336

**Published:** 2022-02-20

**Authors:** Milijana Janjusevic, Alessandra Lucia Fluca, Giulia Gagno, Alessandro Pierri, Laura Padoan, Annamaria Sorrentino, Antonio Paolo Beltrami, Gianfranco Sinagra, Aneta Aleksova

**Affiliations:** 1 Cardiothoracovascular Department, Azienda Sanitaria Universitaria Giuliano Isontina (ASUGI) and Deparment of Medical Surgical and Health Science, University of Trieste, 34149 Trieste, Italy; mjanjusevic@units.it (M.J.); alessandralucia.fluca@units.it (A.L.F.); gagnogiulia@gmail.com (G.G.); alessandro.pierri92@libero.it (A.P.); sorrentinoannamaria135@gmail.com (A.S.); gianfranco.sinagra@asugi.sanita.fvg.it (G.S.); 2 Cardiology and Cardiovascular Physiopathology, Azienda Ospedaliero-Universitaria S. Maria Della Misericordia, 06156 Perugia, Italy; argonauta92@hotmail.it; 3 Department of Medicine (DAME), University of Udine, 33100 Udine, Italy; antonio.beltrami@uniud.it

**Keywords:** hyperglycemia, diabetes mellitus, glucagon-like-peptide-1 receptor agonists (GLP-1RAs), sodium-glucose cotransporter-2 inhibitors (SGLT2i), hypovitaminosis D, endothelial dysfunction, inflammation, atherosclerosis, coronary artery disease, antiplatelets

## Abstract

Hyperglycemia is considered one of the main risk factors for atherosclerosis, since high glucose levels trigger multiple pathological processes, such as oxidative stress and hyperproduction of pro-inflammatory mediators, leading to endothelial dysfunction. In this context, recently approved drugs, such as glucagon-like-peptide-1 receptor agonists (GLP-1RAs) and sodium-glucose cotransporter-2 inhibitors (SGLT2i), could be considered a powerful tool for to reduce glucose concentration and cardiovascular risk. Interestingly, many patients with type 2 diabetes mellitus (T2DM) and insulin resistance have been found to be deficient in vitamin D. Recent studies pointed out the unfavorable prognostic values of T2DM and vitamin D deficiency in patients with cardiac dysfunction, either when considered individually or together, which shed light on the role of vitamin D in general health status. New evidence suggests that SGLT2i could adversely affect the production of vitamin D, thereby increasing the risk of fractures, which are common in patients with T2DM. Therefore, given the biological effects of vitamin D as an anti-inflammatory mediator and a regulator of endothelial function and calcium equilibrium, these new findings should be taken into consideration as well. The aim of this review is to gather the latest advancements regarding the use of antidiabetic and antiplatelet drugs coupled with vitamin D supplementation to control glucose levels, therefore reducing the risk of coronary artery disease (CAD).

## 1. Introduction

Atherosclerosis is one of the major causes of morbidity and mortality worldwide [1]. Atherosclerotic plaques accumulate within the arterial wall of almost every body district and reduce arterial blood flow when a significantly hemodynamic level of stenosis is reached. When atherosclerosis involves the heart, it causes a condition also known as coronary artery disease (CAD). The complication of an atherosclerotic coronary artery with a superimposed thrombotic event leads to the sudden blockage of the affected coronary vessel, an event that severely jeopardizes cardiac perfusion and, if protracted in time, results in irreversible cardiac damage and myocardial infarction [2]. High blood pressure, smoking, advanced age, gender, family history, and metabolic disorders, such as obesity, dyslipidemia, and type 2 diabetes mellitus (T2DM), represent significant risk factors in the development of atherosclerosis and CAD [2,3]. Most of these factors converge in triggering and sustaining both endothelial dysfunction and inflammation, which play an important role in all stages of atherosclerosis, exacerbating disease progression and favoring adverse outcomes [2,3].

In this comprehensive review, we gather current knowledge on the hyperglycemic effects on endothelial cells (ECs), more specifically the glycemic-induced molecular pathways underpinning endothelial dysfunction [4], and the consequences for general health. In addition, we dwell on new therapeutic approaches, with special reference to antidiabetic drugs, such as glucagon-like-peptide-1 receptor agonists (GLP-1Ras) and sodium-glucose cotransporter-2 inhibitors (SGLT2i) [5]. Finally, due to the immense anti-inflammatory and antioxidative roles of vitamin D [6,7], we point out the benefits of cholecalciferol supplementation and the negative consequences in the case of vitamin D deficiency [8,9].

## 2. Molecular Mechanisms of Endothelial Dysfunction

The endothelium plays a key role in cardiovascular homeostasis; protecting against vascular injury; preserving blood fluidity [10]; modulating permeability for nutrients and metabolites; and regulating inflammatory processes, vascular tone, and coagulative processes [11]. Pathological metabolic conditions and risk factors, such as T2DM, obesity, dyslipidemia, and hyperglycemia, which is the main focus of the present review, significantly perturb endothelial functions, including barrier function, vasoregulation, anticoagulant, and anti-inflammatory functions [11,12]. Specifically, hyperglycemia acts on ECs, causing the release of pro-inflammatory cytokines, an increase in adhesion molecules, and the promotion of the pro-thrombotic state. Moreover, functional alterations of ECs have an impact on the extracellular matrix [13,14], vascular smooth muscle cells (VSMCs) [15], and circulating endothelial cells (CECs) [16]. All of these aspects are included in the definition of endothelial dysfunction, which is considered pivotal in the development of both T2DM and CAD and represents a major contributor to cardiovascular events [17].

### 2.1. Effects of Hyperglycemia on the Endothelium

Decreased bioavailability of nitric oxide (NO) is one of the causes of endothelial dysfunction, which could theoretically be a consequence of decreased activity or expression of endothelial nitric oxide synthase (eNOS) and excessive NO degradation [11]. It is important to emphasize that eNOS is a salient component of the insulin-mediated Pi3K/AKT/eNOS pathway. Both in vitro and in vivo studies pointed out that eNOS activity is impaired in the hyperglycemic state [18,19], which explains why hyperglycemia is defined as one of the actors responsible for establishing vascular insulin resistance [12,20].

High glucose concentration causes an increase in diacylglycerol synthesis, which leads to the activation of specific isoforms of protein kinase C (PKC) in ECs [19,21]. PKC is an enzyme involved in a large number of intracellular signaling, and, most importantly in this context, its activity and expression are enhanced in the vascular tissue of patients with T2DM [21]. Specifically, three isoforms of PKC, α, β, and δ, are involved in diabetic vascular complications [21]. The PKC-mediated eNOS phosphorylation on Thr497, which is recognized as a negative regulatory site, reduces enzyme activity [22]. However, the overall underlying mechanisms of PKC-mediated phosphorylation of eNOS are poorly understood and controversial [18]. Multiple studies have demonstrated that PKC inhibitors reverse the decrease in eNOS activity and glucose-induced endothelial dysfunction [18,21,22,23].

**Figure 1:**
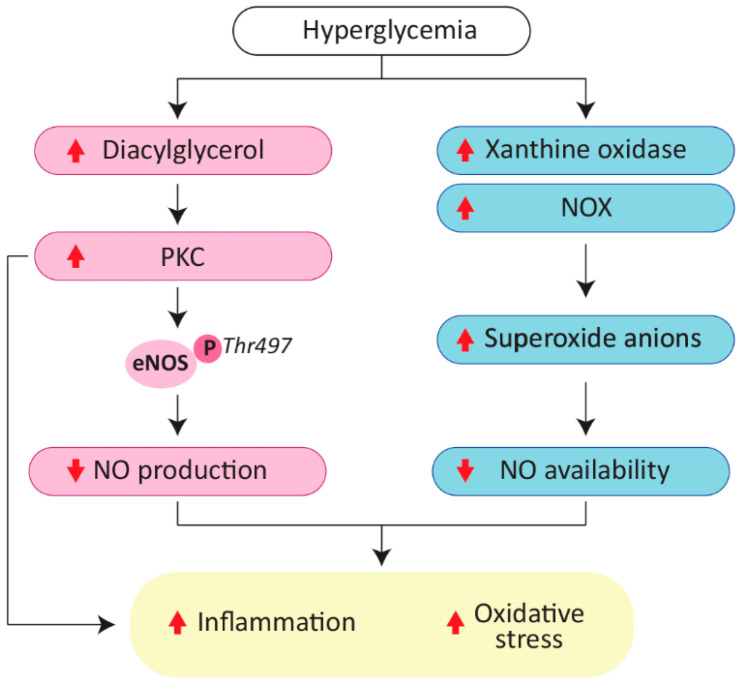
The molecular effects of hyperglycemia, which increases protein kinase C (PKC) activity and therefore enables phosphorylation of endothelial nitric oxide synthase (eNOS) on Thr497, which reduces nitric oxide (NO) production. Moreover, hyperglycemia causes an increase in superoxide anion production, leading to lower NO availability. Inflammation and oxidative stress increase due to NO deficiency. eNOS, endothelial nitric oxide synthase; NO, nitric oxide; NOX, nicotinamide adenine dinucleotide phosphate oxidase; PKC, protein kinase C.

In addition, evidence suggests that NO levels are not sufficient to guarantee endothelial functionality due to eNOS uncoupling, namely because of the production of superoxide anions [18,24]. Indeed, the increase in intracellular glucose concentration mediates the variation of the redox potential and the production of superoxide anions via nicotinamide adenine dinucleotide phosphate oxidase (NOX) and xanthine oxidase, which contribute both to reducing NO availability and promoting the production of peroxynitrite, a strong oxidant (Figure 1) [11,25,26].

The reduction in NO bioavailability has multiple effects. It enhances the action of the nuclear factor kappa-light-chain-enhancer of activated B cells (NF-κB), which induces the synthesis of pro-inflammatory cytokines, such as interleukin (IL)-1β, IL-6, and tumor necrosis factor alpha (TNF-α) [26]. This pro-inflammatory milieu stimulates the endothelial expression of adhesion proteins, leading to the recruitment of immune cells [27,28] with mechanisms that involve either NO or PKCβ. Specifically, as suggested by Tabit et al. [23], in patients with T2DM, PKCβ activity is linked to the activation of NF-κB in ECs. Besides the contribution to the worsening of inflammation, pro-inflammatory cytokines lead to the establishment of a pro-coagulant state, as they promote the expression of tissue factor, plasminogen activator inhibitor-1, and von Willebrand factor [26].

Importantly, the effects of NO are not limited to ECs. Since NO mediates the relaxation of VSMCs [29], in the case of T2DM, the misbalance in NO and vasoconstrictors, such as endothelin, contributes to the impairment of vascular tone and blood flow [15]. Alterations in blood flow cause endoplasmic reticulum stress and downregulation of endothelial cadherin, which lead to apoptosis [16,30]. In this context, CECs may play a relevant role as circulating cells that repair the sites of endothelial injury. However, in patients with T2DM, the amount and functionality of CECs are reduced, which contributes to evident alterations in the reparation process [16].

**Figure 2:**
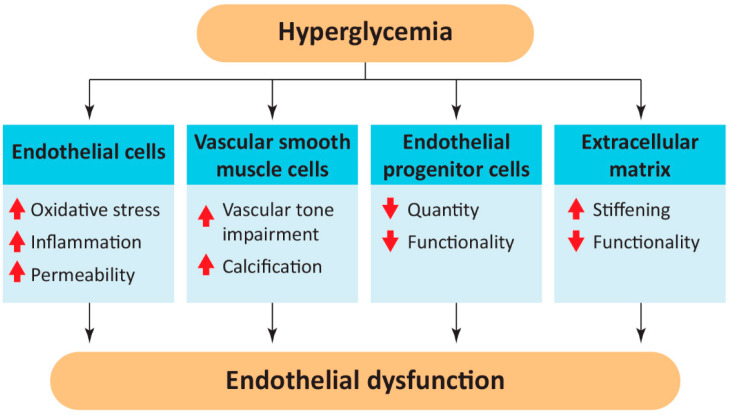
The cumulative effects of high glucose concentration on endothelial cells, vascular smooth muscle cells, circulating endothelial cells, and extracellular matrix that lead to endothelial dysfunction.

NO deficiency is not the only mechanism by which hyperglycemia exerts pathological complications on vessels. Excess glucose is responsible for irreversible glycation of protein, thus producing advanced glycation end products (AGEs), which have a pathological effect not only on ECs due to oxidative stress and protein dysfunction but also on other endothelial components [31]. In particular, AGEs cause vascular calcification, possibly promoting VSMCs senescence and a switch from a contractile to a secretory phenotype [25,32,33]. Furthermore, hyperglycemia contributes to the impairment of the extracellular matrix of blood vessels through the regulation of transcription and activation of metalloproteinases (MMPs). Specifically, the expression and activation of MMP-2 and MMP-9 has been shown to be regulated by oxidative stress and reactive oxygen species (ROS) [13]. Moreover, glycation of proteins of the extracellular matrix compromises protein functionality and contributes to vessel stiffening [13,14]. Furthermore, hyperglycemia is associated with the upregulation of the renin–angiotensin–aldosterone system (RAAS) [34]. High glucose concentrations enhance the transcription of angiotensinogen and the synthesis of angiotensin II (Ang II) by the angiotensin-converting enzyme [34]. Both angiotensinogen and Ang II inhibit the insulin-dependent signaling pathways in ECs and VSMCs, thus aggravating the pro-inflammatory state, accelerating arterial stiffening, and contributing to insulin resistance [34,35]. Importantly, Ang II also increases oxidative stress via NOX [36]. Figure 2 summarizes the effects of hyperglycemia and its contribution to endothelial dysfunction.

### 2.2. From Pre-Diabetes to Diabetes Mellitus towards Coronary Artery Disease

**Figure 3:**
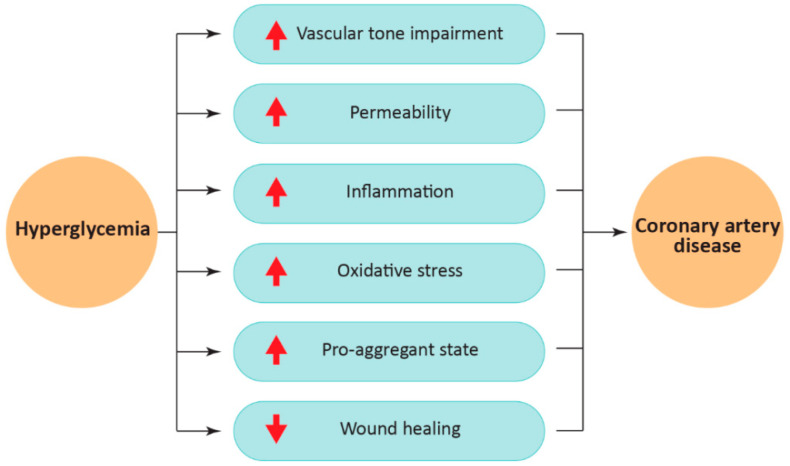
A schematic representation of how hyperglycemia-induced processes affect the pathogenesis of coronary artery disease.

The American Diabetes Association guidelines allow for discrimination of healthy individuals from pre-diabetic and diabetic patients using four diagnostic methods: fasting plasma glucose test, oral glucose tolerance test, glycated hemoglobin, and random plasma glucose [37]. The pre-diabetic condition is generally asymptomatic but often evolves into T2DM as plasma glucose concentrations tend to increase [38]. T2DM and CAD share common lifestyle risk factors and genetic underpinnings [39]. As previously introduced, patients with T2DM exhibit more severe coronary atherosclerosis than non-diabetic individuals [40]. Since chronic hyperglycemia supports endothelial dysfunction, T2DM accelerates the development of severe CAD-associated complications [17]. The overall T2DM-induced alterations, which include dysregulation of vascular tone, increased permeability, inflammation, oxidative stress, and the pro-coagulant state, contribute to the initiation, progression, and degeneration of atherosclerotic plaques [41] (Figure 3).

First, arterial stiffness and calcification cause impaired and turbulent blood flow, which increases the risk of plaque formation [42]. Second, the increase in endothelial permeability favors both the accumulation of low-density lipoprotein (LDL), following their binding to proteoglycans in the tunica intima, and the recruitment of immune cells as monocytes [20]. Third, the pro-oxidant environment induced by hyperglycemia through AGEs and ROS causes the oxidation of LDL, which is easily phagocytized by macrophages, which assume a peculiar foam cell appearance [43]. Oxidative stress, inflammation, and AGEs, in synergy, participate in the alteration of the extracellular matrix and lead to the formation of a fibrous cap [20]. The alterations of the extracellular matrix stimulate the VSMCs to switch from a contractile to a synthetic phenotype, which is triggered for a reparative purpose [44]. However, this mechanism is considered a pathological hallmark of atherosclerosis since it could exacerbate the inflammatory process [44]. As its evolution progresses, the atherosclerotic plaque develops a necrotic core, eventually surrounded by calcified tissue [45]. At a later stage, the plaque is more unstable and prone to rupture due to thinning of the fibrous cap following the apoptosis of VSMCs and the release of MMPs [20]. As previously elucidated, the pro-thrombotic state in patients with T2DM often evolves into thrombus formation, either as a consequence of endothelial erosion or following plaque rupture and exposure of its content in the lumen of blood vessels [26,46,47].

## 3. Therapeutic Approaches

### 3.1. Traditional Antidiabetic Drugs

Metformin is one of the most used antidiabetic drugs and is still recommended as a first-line therapy for T2DM [48]. The hypoglycemic effect is mainly due to a stimulation of insulin synthesis, resulting in a reduction in hepatic glucose release and in an increase in peripheric glucose uptake, which leads to a decrease in insulin resistance [49]. However, the risk of hypoglycemia is very low during metformin treatment [50]. Moreover, this drug has few adverse effects, the most serious of which is lactic acidosis. However, the increase in blood lactate levels represents a rare complication, even among patients with predisposing factors, such as chronic kidney disease [50,51].

Apart from glycemic control, metformin possesses several additional proprieties that have a positive impact on endothelial function, such as lipidic control and an anti-inflammatory effect through a reduction in oxidative stress, [48,52]. Furthermore, there is some evidence derived from in vitro and animal studies suggesting that metformin might lower blood pressure and counteract the effects of Ang II on cardiomyocytes [53]. Other interesting metformin effects are related to cardiac ischemic damage, as it contributes to reduced infarct size in patients with T2DM, thanks to a more efficient shift from fatty acid aerobic oxidation to glucose anaerobic utilization [54]. Moreover, metformin treatment, compared to other hypoglycemic drugs, resulted associated to a better outcome after myocardial infarction in patients with T2DM [55]. Part of these positive effects may be mediated by AMP kinase (AMPK). This protein kinase regulates cell metabolism and promotes the activation of autophagy, a mechanism that has been associated with myocardial regeneration in zebrafish [56].

An emerging mechanism of action of metformin relies on the modulation of the gut microbiome. Indeed, the drug is more effective when administered orally, is found at very high concentrations in the small intestine, and, in a relevant proportion of cases, may also be found in the large intestine [57]. Furthermore, studies conducted on patients affected by T2DM have shown that metformin may revert their altered microbiome by increasing the butyrate-producing taxa [58]. Intriguingly, fecal transplantation from metformin-treated patients with T2DM to germ-free mice fed with a high-fat diet improved glucose tolerance in the transplanted animals [59].

Finally, many animal studies have reported a positive effect of metformin on left ventricular remodeling, although there is a lack of data about this effect on humans [55,60]. In patients with both CAD and T2DM, metformin reduces cardiovascular mortality, all-cause mortality, and cardiovascular events, probably sustaining endothelial protection through its hypoglycemic and anti-inflammatory properties [61].

Thiazolidinediones (TZDs) are effective for achieving good glycemic control in patients with T2DM. This class of drugs acts to improve insulin sensitivity through interaction with the peroxisome proliferator-activated receptor gamma (PPAR-γ) [62,63]. Beyond this effect on glycemic control, TZDs are responsible for various positive cardiovascular effects, as they contribute to lowering blood pressure through multiple mechanisms, have a positive impact on lipid profile and endothelial function, and reduce inflammation [62]. Given all the effects, TZDs could contrast atherosclerosis progression, which has been reported both in animal models and humans [63]. Some studies noted a positive impact of TZDs on cardiovascular outcomes [62]. However, other works reported a higher incidence of heart failure among patients treated with TZDs [62]. For this reason, TZDs are currently contraindicated in patients with symptomatic heart failure.

### 3.2. New Antidiabetic Drugs

Recently, new medications initially developed to treat diabetes, namely GLP1RAs and SGLT2i, have been proven to have beneficial effects on the cardiovascular system partially independently of glycemic control.

#### 3.2.1. Glucagon-like-Peptide-1 Receptor Agonists

Glucagon-like peptide-1 (GLP-1) is an incretin hormone produced and released by gut enteroendocrine cells in response to glucose ingestion. It exerts its effects after binding to the GLP-1 receptor (GLP-1R), which has several functions, including delaying gastric emptying and reducing food intake through increased satiety [64]. In addition, GLP-1 stimulates insulin production and inhibits glucagon secretion, thus playing a key role in controlling glycemic excursion after meals. Furthermore, it contributes to the reducing oxidative stress and inflammatory response [65]. Given these beneficial effects, some agonist drugs have been developed that either simulate GLP-1 effects through GLP-1R (i.e., the previously mentioned GLP-1RAs) or by preventing GLP-1 degradation via the inhibition of dipeptidyl peptidase-4 (DPP-4) [66].

GLP-1RAs have proven efficacy and a good safety profile for the treatment of patients with T2DM [67]. GLP-1RAs can be divided into two groups, depending on their “short” or “long” activity, the latter having demonstrated a greater power in reducing glycated hemoglobin. Apart from being effective in the treatment of T2DM, the administration of GLP-1RAs has also proven beneficial in terms of body weight reduction. The mechanisms of this body weight reduction are not yet known, but it is likely that both central and peripheral mechanisms are involved [67]. Furthermore, several trials have demonstrated either non-inferiority or superiority for cardiovascular outcomes of treatment with these drugs in patients with T2DM [68]. In a recent meta-analysis of such trials, treatment with GLP-1RAs was associated with a 12% reduction in major adverse cardiac events and all-cause death, as well as a 9% reduction in hospitalization for heart failure [69]. As T2DM is one of the major cardiovascular risk factors, on one hand, it is likely that GLP-1RAs exert a beneficial effect on the cardiovascular system in an indirect way by lowering glucose levels, thus reducing cardiovascular risk. On the other hand, since not all of the aforementioned beneficial effects of GLP-1RAs can be explained by decreased glucose levels, it has been suggested that stimulating the GLP-1R can also have direct beneficial effects on the cardiovascular system, for example, by contrasting the progression of atherosclerosis [70]. Specifically, GLP-1RAs reduce the production of inflammatory cytokines and leucocyte recruitment, regulate atherosclerotic plaque stability through the control of MMPs acting on the extracellular matrix, and by suppressing the VSMCs switch, regulate vascular remodeling [70,71]. This idea is in line with preclinical studies wherein native GLP-1 has been found to ameliorate endothelial function and improve myocardial glucose uptake and ventricular contractility, thus having a protective effect on blood vessels and cardiomyocytes [66]. In the cardiovascular system, the beneficial effects of GLP-1, its degradation products, and GLP-1RAs have been confirmed in human studies [66]. Given all these positive effects of GLP-1RAs on cardiovascular outcome, these drugs have been listed among class I treatment in patients with T2DM and atherosclerotic cardiovascular disease by most recent guidelines [72].

In the same context, it is worth mentioning the positive effect of DPP-4 inhibitors (DPP-4is) on endothelial function. In a recent meta-analysis, it was pointed out that DPP-4i significantly reduced glycemic variability and prevent further complications in comparison to other antidiabetic drugs among patients with T2DM [73]. DPP-4 improves glucose and glycemic control in patients with T2DM by preserving GLP-1 and β-cells by inhibiting apoptotic pathways [73]. Dell’Oro et al. showed that in a small cohort of patients with T2DM who failed to control glycemic levels with metformin, one year of saxagliptin treatment yielded positive results in glycemic control and improved endothelial function [74]. By controlling hyperglycemia, saxagliptin has a positive effect on the endothelium because it consequently regulates glucose-induced inflammation and oxidative stress.

#### 3.2.2. Sodium-Glucose Cotransporter-2 Inhibitors

SGLT2i, also called gliflozins, represent an emerging drug class with proven beneficial effects at the level of the kidneys and cardiovascular system. These agents act directly on sodium-glucose cotransporter 2 (SGLT2), which is mainly located in the proximal tubule of the kidney and mediates the vast majority of renal glucose reuptake. By blocking glucose reabsorption, SGLT2i enhances urinary glucose excretion, thereby diminishing glycemic levels [75].

Although SGLT2i were originally been adopted as oral hypoglycaemic agents for the treatment of T2DM, recent evidence has demonstrated that their therapeutic benefits go beyond better glycemic control [76]. Focusing on the cardiovascular system, different cardioprotective mechanisms have been proposed to explain the efficacy of SGLT2 inhibition in reducing the risk of cardiovascular death and hospitalization as a result of heart failure both in patients with and without T2DM, as confirmed by numerous clinical trials [77,78,79,80,81,82].

Specifically, the increasing of diuresis/natriuresis [83,84] with favorable hemodynamic effects, the improvement of cardiac energy metabolism [85,86], the inhibition of both the sympathetic nervous system [87] and the cardiac Na^+^/H^+^ exchanger [88] appear to be the most important components of the cardioprotective activity of gliflozines. Furthermore, SGLT2i exhibit significant anti-inflammatory properties, potentially attenuating the molecular and cellular pathways that result in atherosclerosis. Indeed, the fact that inflammation constitutes the key pathophysiological feature of atherogenesis fuels the assumption that SGLT2i may counteract the atherosclerotic process, which is, in turn, a major contributor to the vascular complications of T2DM, including CAD. Reliable experimental studies have shown that gliflozins are able to mitigate atherosclerosis progression in animal models through multifaceted mechanisms of action. In particular, in non-diabetic mice, empagliflozin reduces LDL cholesterol (LDL-C) levels [89], which are conspicuously related to atherosclerosis, whereas in patients with T2DM, dapagliflozin decreases small dense LDL-C in comparison with sitagliptin [90]. Additionally, SGLT2i reduce myocardial oxidative stress in diabetic mice by decreasing ROS production [91] and improve endothelial function, especially by restoring NO bioavailability [92]. Apart from these beneficial effects, gliflozins contribute to the prevention of the atherosclerotic process by inhibiting the transformation of macrophages into foam cells [93], reducing plaque size and burden [89,94], and lowering blood pressure [95]. Nevertheless, the antihypertensive effect of SGLT2i is quite modest, probably covering a secondary role in protection against atherosclerosis.

A well-recognized target of SGLT2i is represented by nucleotide-binding domain-like receptor protein 3 (NLRP3) inflammasome. The activation of this multiprotein complex plays a key role in both the development and progression of many diseases sustained by inflammation, such as atherosclerosis [96]. Notably, NLRP3 inflammasome promotes IL-1β secretion through a two-step pathway involving caspase-1 activation, triggering vascular inflammation and contributing to the progression of atherosclerotic lesions [97,98]. Interestingly, gliflozins have been identified as potent NLRP3 inflammasome inhibitors, enhancing their cardioprotective effects. In a recent study conducted to assess the capacity of SGLT2i to attenuate NLRP3 inflammasome activation, Kim et al. found that SGLT2i caused a significant reduction in IL-1β production compared to sulfonylureas in patients with T2DM and high cardiovascular risk [99]. This effect was predominantly attributed to the increased serum concentration of β-hydroxybutyrate, which was demonstrated to suppress NLRP3 inflammasome activation in previous ex vivo experiments [100].

The impact of SGLT2i in the specific setting of ischemic heart disease has been partially investigated. In a retrospective study, Lee et al. revealed that SGLT2i improve cardiovascular function in patients with T2DM and CAD compared to DPP-4i [101]. Besides, based on cardiac magnetic resonance imaging, the EMPA-HEART CardioLink-6 trial showed a significant reduction in myocardial extracellular compartment volume and indexed left ventricular mass in a cohort of diabetic patients with CAD after six months of treatment with empagliflozin [102]. Returning to the animal model, a beneficial effect on coronary microvascular dysfunction was detected by Adingupu et al. in prediabetic mice treated with SGLT2i [103]. As discussed by the authors, this effect was likely related to the NO-dependent improvement of endothelial function, as well as better glycemic control.

Based on the broad spectrum of nephro- and cardioprotective mechanisms, the application of SGLT2i in clinical settings is likely just beginning.

#### 3.2.3. Pro-Thrombotic State in Hyperglycemia and Pharmacological Intervention

Hyperglycemia determines direct vascular damage through the enhanced production of intracellular AGEs and the activation of the PKC pathway, which triggers platelet activation [104]. Additionally, hyperglycemia leads to the prothrombotic state due to the increased expression of procoagulant factors, such as plasminogen activator inhibitor-1 (PAI-1) [105,106]. Moreover, hyperglycemia causes platelet dysfunction and hyperreactivity through multiple mechanisms. First, the glycation of proteins on the surface of platelets increases their adhesion. Second, the enhancement of glycoprotein IIb/IIIa activation and P-selectin expression contributes to platelet stimulation and adhesion [106]. Third, a decreased response to antithrombotic mediators, such as prostacyclin (PGI2) and NO, occurs [105,107]. Finally, hyperglycemia can also act indirectly through the glycation of circulating LDL, which results in NO hypoproduction and intraplatelet calcium hyperconcentration [107,108].

Many studies have suggested that platelets produce and harbor large amounts of microRNAs (miRNAs) and represent a major source of circulating miRNAs in the blood [109,110]. The same studies demonstrated that hyperglycemia is implicated in the expression of these microRNAs in diabetic patients and that these molecules, such as miR-223, have a role in the regulation of platelet activation. In particular, there is evidence that patients with T2DM exhibit significantly lower levels of miR-223 induced by hyperglycemia, which results in anomalies in platelet function [109,110,111]. It is worth mentioning that in patients with T2DM, significantly lower levels of miR-223 lead to the deregulation of the VSMCs switch, a hallmark of atherosclerosis [112]. Additionally, other miRNAs known to play a role in maintaining normal endothelial function have been shown to have deregulated levels in patients with T2DM [113].

Furthermore, it has been noted that diabetic patients present an augmented mean platelet volume and increased oxidative stress due to excessive production of ROS, determined by the activation of NOX2 [114].

For all these reasons, patients with T2DM or hyperglycemia are scarcely responsive to antiplatelet therapies. Specifically, although there is strong evidence of an aspirin-mediated response to weak agonists, such as adenosine diphosphate (ADP), aspirin has limited effects on potent agonists, such as thrombin [107]. In addition to its antiplatelet proprieties, aspirin has been shown to improve glucose tolerance due to its anti-inflammatory properties and through incrementation of NO levels, which facilitates insulin signaling [115,116]. However, there is no current evidence that aspirin prevents T2DM [107].

Aspirin is not the only antiplatelet drug that has been evaluated for the treatment of patients with T2DM. Several studies have demonstrated that the P2Y12 inhibitors improve the effects of NO [117]. Among the P2Y12 inhibitors, clopidogrel is less effective than ticagrelor and prasugrel in preventing cardiac events in patients with acute coronary syndrome [118]. Prasugrel provides the strongest effect on ADP-induced platelet aggregation, and for this reason, its use should be favored among diabetic patients with acute coronary syndrome [119,120]. Because aspirin alone is not sufficient for patients with CAD, the administration of multiple antiplatelet drugs has been investigated, although its feasibility remains questionable [107]. Patients treated with aspirin and ticagrelor or prasugrel showed an increase in plasma miR-223 concentration, and this correlates with a reduction in ADP-induced platelet activation. Therefore, it seems that miR-223 expression may reflect the level of platelet inhibition during P2Y12 inhibitor therapy [121]. Additionally, an inhibitory activity of ticagrelor on NLRP3 inflammasome activation via AMPK has been described [122].

In the recent years, several studies have tried to determine whether antidiabetic drugs have an impact on platelet function. Metformin reduces platelet activity and controls oxidative stress [123]. Additionally, therapy with metformin has been shown to be associated with a reduction in mean platelet volume [124]. Furthermore, treatment with TZDs such as pioglitazone seems to be associated with higher inhibition of platelet aggregation than treatment with glipizide, a sulfonylurease, due to the underexpression of P-selectin. However, glipizide appears more effective in controlling the concentration of glucose in the blood [125]. DPP4is have shown potential beneficial effects on oxidative stress and endothelial function. In particular, they reduce the expression of thrombogenic genes and implement the mitochondrial function of platelets [126].

In addition, the recent antidiabetic drugs GLP1-RAs and SGLT2i seem to have positive effects. Specifically, since the GLP-1R is present on platelets, its activation through GLP1-RAs leads to a reduction in ADP-induced platelet aggregation [127]. SGLT2i, such as empagliflozin, canagliflozin, and dapagliflozin, which recently emerged as central therapies for heart failure, even in the absence of T2DM, have both direct and indirect antithrombotic effects, mostly mediated by enhanced NO and PGI2 production [128]. Therefore, apart from the improvement of inflammatory state and endothelial dysfunction, SGLT2i reduce platelet aggregation and AGE production [129,130,131].

Finally, there is evidence that hypovitaminosis D is associated with a pro-thrombotic state due to its negative effects on endothelial function, NO levels, and inflammation [132]. Vitamin D deficiency appears to have a direct effect on platelets, as it causes increased platelet aggregation [133] and ADP-mediated platelet activation [134]. Moreover, vitamin D receptor (VDR) is expressed on the mitochondria of platelets [135]. These facts are of utmost importance since vitamin D deficiency is common among patients with T2DM, and both vitamin D deficiency and T2DM have a negative impact on the prognosis of patients with cardiovascular disease [136]. Since SGLT2i have a potentially negative impact on bone health and vitamin D levels [137], vitamin D supplementation may be a useful weapon for avoiding the adverse effects of SGLT2i, concomitantly counteracting platelet dysfunction among patients with T2DM. Table 1 summarizes the effects of all drugs mentioned in these paragraphs.

## 4. Vitamin D and Its Association with Cardiovascular Diseases

In the same context, it is extremely important to mention the association of vitamin D deficiency with the risk of developing cardiovascular disease coupled with T2DM and insulin resistance, as well as the correlation with disease severity [138,139]. Epidemiological studies have demonstrated that low levels of vitamin D correlate with cardiovascular diseases and associated unfavorable outcomes [8,9]. A large amount of experimental evidence has indicated a correlation between vitamin D deficiency and decreased insulin secretion, and it has been observed that it may also influence glucose intolerance [138,140]. Vitamin D deficiency is common in patients with T2DM and is associated with disease complications [136]. Moreover, a study involving patients with atherosclerosis and T2DM demonstrated that lower concentrations of vitamin D were associated with a higher number of stenotic coronary arteries [141]. Furthermore, our study involving patients surviving a myocardial infraction pointed out the prognostic power of vitamin D in terms of unfavorable outcomes, both alone and in combination with the presence of T2DM [136].

Up to about 90% of vitamin D is synthesized in the skin after exposure to sunlight, whereas a small amount comes from food [142]. Vitamin D exerts its genomic actions by binding to the VDR, which is found in various tissues and organs, such as cells of the immune system, heart, ECs, pancreatic islets, liver, kidney, and gastrointestinal tract [143,144]. A growing body of evidence has pointed out that vitamin D directly or indirectly controls more than 3% of the human genome, including genes involved in the regulation of cell proliferation, differentiation, DNA repair, and apoptosis [143,145,146]. Vitamin D performs its non-genomic actions, thanks to the transmembrane second messenger systems, including mitogen-activated protein kinases (MAPK), protein kinase A (PKA), Akt, PKC, and proto-oncogene tyrosine-protein kinase Src [144,147]. Therefore, apart from its role in the regulation of calcium homeostasis and bone health [148], nowadays it is known that vitamin D is involved in many processes, such as the modulation of the immune response, antioxidant roles, and participation in glucose homeostasis [149].

**Figure 4:**
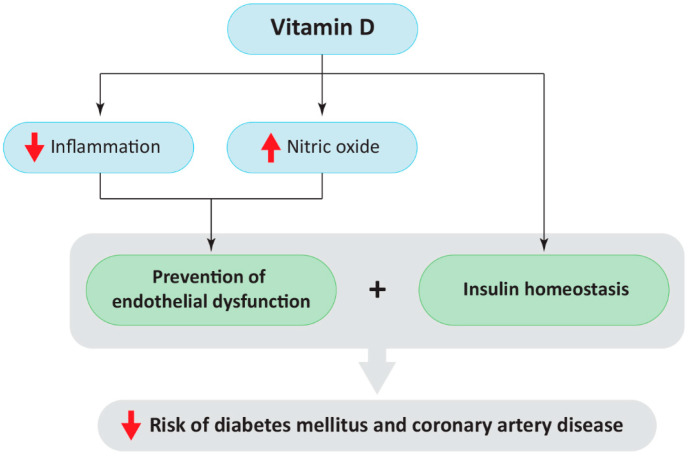
Summary of the positive effects of vitamin D on insulin homeostasis, inflammation control, and nitric oxide production to prevent endothelial dysfunction.

The association between vitamin D deficiency and the pathophysiology of cardiovascular disease can be explained on several levels (Figure 4). Vitamin D promotes the anti-inflammatory state by suppressing the activity of the NF-κB pathway, thus reducing the concentration of pro-inflammatory cytokines, such as IL-1β, IL-6, and TNF-α [6,7]. Additionally, vitamin D has been found to enhance eNOS, therefore stimulating NO production. In the same context, vitamin D reduces ROS production and H_2_O_2_ oxidative stress. Therefore, in cases of vitamin D deficiency, its protective roles are impaired, leading to endothelial dysfunction, which is the first step towards atherosclerosis and myocardial infarction. [6]. Moreover, emerging data has pointed out that vitamin D is involved in RAAS regulation. Specifically, vitamin D suppresses *renin* gene expression through a vitamin D-responsive element in the promoter of the *renin* gene and *angiotensin* gene expression by blocking the NF-κB pathway [6]. Therefore, vitamin D is involved in the regulation of blood pressure via RAAS. Additionally, vitamin D regulates glucose homeostasis by stimulating insulin synthesis, as well as insulin secretion indirectly through the regulation of calcium concentration in plasma and directly by interacting with pancreatic beta cells [138]. In vitro and in vivo models showed that vitamin D is required for the normal release of insulin in response to glucose and that vitamin D may be essential for the preservation of glucose tolerance [138]. Therefore, in the case of vitamin D deficiency, those processes are impaired.

As noted previously, patients with T2DM are prone to the development of CAD and myocardial infarction over time since a high concentration of glucose leads to an increase in oxidative stress and inflammation and therefore endothelial dysfunction and atherogenesis [150]. Moreover, as mentioned above, hyperglycemia stimulates RAAS and the expression of pro-inflammatory cytokines. Thus, the cumulative effects of hyperglycemic status and vitamin D deficiency, which share similar findings, explain the complications of the disease in patients with T2DM and vitamin D deficiency [136]. These claims are supported by studies that have shown that higher levels of vitamin D correlate with a lower risk of developing T2DM [151,152] and that vitamin D supplementation improves glycemic control in patients with T2DM [153] and possibly reduces the risk of T2DM in patients in the pre-diabetic phase [154,155]. It is important to mention that there is an inconsistency regarding vitamin D supplementation studies and the benefits introduced above. A meta-analysis by Seida et al. encompassing 35 trials pointed out no beneficial effects of vitamin D supplementation on glucose control, insulin resistance, or T2DM prevention [156]. Moreover, multiple studies have shown that vitamin D can prevent heart disease [157,158], whereas several others have failed in this endeavor [159,160]. Possible explanations for this contradiction may be linked to the limitations in study design, such as duration, insufficient or infrequent doses of vitamin D, and inadequate assessment of subjects in terms of lack of verification of whether subjects reached levels of sufficiency after supplementation. In addition, for each subject specifically, it is necessary to determine the right dose of supplementation, considering features such as age, body mass index, and baseline vitamin D levels [161]. A recently published observational analysis on data from 33 studies found a causal relationship between vitamin D concentrations and mortality only in individuals with basal low vitamin D status, whereas this relationship was not detected in individuals with normal levels of vitamin D. These findings suggest that further trials investigating the benefit of vitamin D supplementation should consider basal concentrations of this hormone [162].

Taking into account that vitamin D levels certainly contribute to general health, we recommend sun exposure for 30 min twice a week [163] or vitamin D supplementation in combination with antidiabetic and/or anti-inflammatory drugs.

## 5. Conclusions

Hyperglycemia triggers several pathophysiological molecular mechanisms that support endothelial dysfunction and lead to atherosclerosis and CAD. Namely, hyperglycemia leads to increased oxidative stress, inflammation, decreased availability of NO, impaired vascular tone, increased permeability, and procoagulant state, processes responsible for the initiation, progression, and degeneration of atherosclerotic plaques. Multiple studies have shown that implementation of antidiabetic drugs, such as GLP-1RAs and SGLT2i, interrupt or mitigate several processes involved in the development of atherosclerosis, thereby leading to a reduction in cardiovascular complications, disease development and progression, and adverse cardiovascular outcomes. In addition, considerable number of patients with T2DM or insulin resistance were found to be deficient in vitamin D. Recent studies have shown a powerful predictive and prognostic value of this steroid hormone, in addition to its multiple regulatory roles in processes such as inflammation, oxidative stress, glucose homeostasis, and insulin resistance. Therefore, along with medications usually recommended against hyperglycemia, we strongly suggest vitamin D supplementation.

## Figures and Tables

**Table 1:** Summary of the effects of antidiabetic drugs, anti-inflammatory drugs, P2Y12 inhibitors, and vitamin D.

Category of Drug	Drugs	Effects	References
Antidiabetic	Metformin	Lower risk of hypoglycemia. Reduced hyperlipidemia. Lower oxidative stress. Regulation of gut microbiome. Reduction in cardiovascular events. Lower mean platelet volume.	[48,49,50,51,53,58,61,123,124].
	Thiazolidinediones	Good glycemic control. Increased insulin sensitivity. Control of blood pressure and hyperlipidemia. Reduced inflammation. Contrast atherosclerosis. Reduction in cardiovascular events. Decrease in P-selectin expression.	[62,63].
	Glipizide	Good glycemic control.	[125]
	Dipeptidyl peptidase-4 inhibitors	Reduced glycemic variability. Glucose and glycemic control. Lower oxidative stress. Ameliorated endothelial function. Lower expression of thrombogenic genes. Proper mitochondrial function.	[73,74,126]
	GLP1-RAs	Insulin production. Reduced glucose concentration. Lower oxidative stress and inflammation. Body weight control. Reduced inflammation. Controlled atherosclerotic plaque stability. Reduced cardiovascular and adverse events. Reduced ADP-induced aggregation. Inhibited P-selectin expression	[65,67,68,70,71,127]
	SGLT2i	Hemodynamic control. Lower oxidative stress and inflammation. Controlled atherosclerotic plaque stability. Reduced hyperlipidemia. Enhanced NO and PGI2 production. Reduced platelet aggregation. Lower AGE production. Negative effects on bone health.	[85,89,91,92,128,129,130,131]
Anti-inflammatory	Aspirin	Antiplatelet and anti-inflammatory properties. Increased NO levels.	[107,115,116]
P2Y12 inhibitors	Ticagrelor	Potentiation of endogenous NO. Increased miR-223. Reduced inflammation.	[119,120,121]
	Prasugrel	Potentiation of endogenous NO. Increased miR-223. Reduction in ADP-induced platelet aggregation.	[119,120,121]
	Clopidogrel	Potentiation of endogenous NO. Lower effectiveness than ticagrelor and prasugrel.	[117]
Steroid	Vitamin D	Reduced SGLT2i adverse effects. Reduced inflammation. Ameliorated endothelial function and NO levels.	[133,134,135,137]

## Data Availability

Not applicable.
